# Efficacy and safety of guselkumab in European patients with palmoplantar pustulosis: A multi-center, single-arm clinical trial (GAP study)

**DOI:** 10.1016/j.jdin.2024.09.001

**Published:** 2024-10-02

**Authors:** Dagmar Wilsmann-Theis, Selina Patt, Andreas Pinter, Sascha Gerdes, Nina Magnolo, Robert Németh, Jennifer Schmitz, Cornelia Paul, Matthias Augustin, Petra Staubach, Ansgar Weyergraf, Ulrike Hüffmeier, Kerstin Wolk, Robert Sabat, Rotraut Mößner

**Affiliations:** a Department of Dermatology and Allergology, University Medical Center Bonn, Bonn, Germany; b Department of Dermatology, Venereology and Allergology, University Hospital Frankfurt am Main, Frankfurt am Main, Germany; c Department of Dermatology, Venereology and Allergology, University Hospital Schleswig-Holstein, Campus Kiel, Kiel, Germany; d Department of Dermatology, University Hospital Münster, Münster, Germany; e Institute for Medical Biometry, Informatics and Epidemiology, University Hospital Bonn, Bonn, Germany; f Clinical Study Core Unit Bonn, Institute of Clinical Chemistry and Clinical Pharmacology, University Bonn, Bonn, Germany; g Institute of Health Care Research in Dermatology and Nursing, University Medical Centre Hamburg-Eppendorf, Hamburg, Germany; h Department of Dermatology, University Medical Center, Mainz, Germany; i Outpatient and Studycenter on the Hase Gbr, Bramsche, Germany; j Institute of Human Genetics, Universitätsklinikum Erlangen, FAU Erlangen-Nürnberg, Erlangen, Germany; k Psoriasis Research and Treatment Center, Department of Dermatology, Venereology and Allergology & Institute of Medical Immunology, Charité - Universitätsmedizin Berlin, Berlin, Germany; l Department of Dermatology, University Medical Center Göttingen, Göttingen, Germany

**Keywords:** guselkumab, IL-23, interleukin-19, multicenter trial, palmoplantar pustular psoriasis, palmoplantar pustulosis, PPPASI, smoking

## Abstract

**Background:**

Palmoplantar pustulosis (PPP) is a chronic inflammatory skin disorder that affects palms and soles. Patients suffer significant pain, itching, and daily activity impairment. Guselkumab, an interleukin-23 inhibitor, has been approved for PPP treatment in Japan. However, there is no effective therapy licensed for PPP in Europe and the USA.

**Objective:**

To explore the efficacy and safety of guselkumab in patients with moderate-to-severe PPP in the Caucasian population.

**Methods:**

A multicenter, single-arm, phase II study involving 50 patients with moderate-to-severe PPP treated with 100 mg guselkumab subcutaneously for 24 weeks was conducted (GAP). Primary endpoint was the reduction of palmoplantar-pustulosis psoriasis area and severity index (PPPASI) at week 24 compared to baseline. Secondary endpoints included physician-assessed and patient-reported measures. Serum samples were taken for exploratory studies.

**Results:**

The primary endpoint was met with a significant median PPPASI reduction by 59.6% at week 24 compared to baseline (*P* < .001). The proportions of patients achieving PPPASI-50 and PPPASI-75 at week 24 were 66.0% and 34.0%, respectively. Median dermatology life quality index dropped from 15 at baseline to 5 at week 24 (*P* < .001). Week 4 changes in interleukin-19 serum levels predicted week 24 clinical response.

**Conclusion:**

Guselkumab may be a promising therapeutic option for PPP in Caucasian patients.

**Capsule Summary**

• Guselkumab has been widely used for psoriasis and increasingly is seen as an option for palmoplantar pustulosis.
• Interleukin-19 may be a predictive biomarker for the therapy response in palmoplantar pustulosis.

## Introduction

Palmoplantar pustulosis (PPP) is a chronic inflammatory skin disease that manifests as erythematous scaly and crusty lesions with numerous pustules on the palms and/or soles. Due to pruritus, burning sensation and pain, patients suffer from severe functional impairment, with a strong impact on their quality of life.^1^^,^^2^

Due to the overlap of clinical features with psoriasis vulgaris (PV), it has been debated whether PPP is a subtype of psoriasis.^2^^,^^3^ However, the genetics of PPP and PV differ significantly.^2^^,^^4^ The pathogenesis of these 2 diseases also appears to overlap only partially.^5^ PV is understood as an excessive reaction of tissue cells, especially keratinocytes, to an ongoing immune activation within the skin, which is dominated by the so-called interleukin (IL)-23 pathway.^4^ In addition to the proximal mediator IL-23, which acts on lymphocytes, this immune pathway includes the more distal cytokines IL-17A, IL-17F, and IL-22, which directly alter the biology of tissue cells.^6^, ^7^, ^8^, ^9^ In PV, the impact of IL-23 pathway cytokines is enhanced by tumor necrosis factor (TNF)-α.^10^ In contrast to PV, the pathogenesis of PPP is only partially understood and different pathogenetic mechanisms have been suggested.^2^^,^^5^^,^^11^ Mediators that cause or enhance the migration of neutrophilic granulocytes into the epidermis or the subsequent activation of these cells play a role.^12^, ^13^, ^14^, ^15^ Additionally, T cells with a transient Th17/2 phenotype seem to be involved in the pathogenesis of PPP.^16^^,^^17^ Interestingly, a significant proportion of patients with PPP are smokers,^18^ and nicotine supports the infiltration and presence of neutrophilic granulocytes in the skin.^19^

The evidence for the use of medical drugs commonly applied in PPP, such as methotrexate, cyclosporine A, and acitretin, is very limited. It appears that these drugs are only moderately effective in this disease and, with long-term use, associated with adverse events.^20^^,^^21^ Furthermore, several biologics, known to be highly effective in PV, such as inhibitors of IL-12/IL-23, TNF-α, and IL-17A,^4^ did not show sufficient efficacy in PPP.^22^, ^23^, ^24^, ^25^ For example, ustekinumab (anti-IL-12/IL-23 monoclonal antibody) and secukinumab (anti-IL-17A monoclonal antibody) failed to reach the primary efficacy endpoint in a small-size and a phase-3b randomized placebo-controlled trial, respectively, in patients with PPP.^23^^,^^24^ Furthermore, spesolimab (anti-IL-36 receptor blocker), a monoclonal antibody approved for the treatment of generalized pustular psoriasis, did not reach the phase-2a randomized placebo-controlled trial primary endpoint in PPP either.^26^ In contrast, a phase-3 clinical trial in Japan with 159 patients with PPP investigating guselkumab, a human IgG1 monoclonal antibody targeting the p19 subunit of IL-23, demonstrated efficacy in PPP.^27^ Based on this, guselkumab has been approved in Japan for PPP treatment. Thus, there is still a very high need for efficient, safe, and well-tolerated therapies for PPP in the rest of the world.

Here, we present results of a single-arm phase-2 study conducted in Germany that investigated the effect of guselkumab on clinical, patient-reported, and immunological parameters in European patients with moderate-to-severe PPP.

## Methods

### Clinical study design and enrolled patients

Study patients were aged 18 years or older and had a diagnosis of PPP for at least 6 months. They had moderate-to-severe PPP defined as palmoplantar pustulosis psoriasis area and severity index (PPPASI) ≥12, were eligible for systemic treatment, and did or did not have concomitant plaque-psoriasis (body surface area <10%). Patients were excluded if they had treatment with IL-23 inhibitor or any therapy targeting TNF-α or IL-17 within the last 3 months, ustekinumab treatment within the last 4 months, other PPP-directed therapies including psoralen-(P)ultraviolet A phototherapy, conventional therapies or apremilast within the last 28 days, or ultraviolet B therapy or topical therapies within the last 14 days before baseline.

Patients received 100 mg guselkumab subcutaneously at week (wk) 0, 4, 12, and 20. Physician-determined clinical and patient-reported parameters were recorded. Moreover, blood samples were taken at baseline and at wk 4, 12, and 24 of treatment for safety evaluation and for exploratory analyses.

The primary endpoint of this study was the reduction (percent change) in PPPASI at wk 24 compared to baseline. Secondary endpoints included the absolute and percent changes in PPPASI and dermatology life quality index over time, the change in pustule count compared to baseline and the change in pain and pruritus over the past week (as determined by a numeric rating scale) relative to baseline. Exploratory analyses included IL-19 serum levels and their association with the therapeutic response.

The trial (EUDRA-CT-No. 2018-004451-20) described has been carried out in accordance with The Code of Ethics of the World Medical Association (Declaration of Helsinki) for experiments involving humans and all procedures were performed in compliance with the relevant laws and institutional guidelines. The trial was approved by the local ethical review boards (17th April 2019, reference number 135/19) and written informed consent was obtained from all study participants.

### Control participants

For exploratory blood analyses, blood from 14 healthy participants (mean age (standard deviation): 37.9 (11.2), female: 57.1%) served as control samples. Sampling and analyses were approved by the institutional review board of Charité - Universitätsmedizin Berlin, Germany (EA2/254/18, Jan 23, 2019), and written informed consent was obtained from all participants.

### Serum biomarker analysis

Serum concentrations of IL-19 were quantified using Quantikine enzyme-linked immunosorbent assay from R&D Systems.

### Statistical analysis

Analyses of primary and secondary endpoints were based on the full analysis set which comprised all patients who received at least 1 dose of the study drug. Missing values were imputed by the usage of baseline observation carried forward, if not indicated otherwise. Statistical comparison between treatment and baseline values was done based on the Wilcoxon signed-rank test (two-sided). Correlation was investigated by Spearman’s correlation analysis. The influence of baseline characteristics on the outcomes was explored by means of logistic regression models (binary outcomes) and by mixed model for repeated measures. *P* values <.05 indicate significance.

## Results

**Fig 1:**
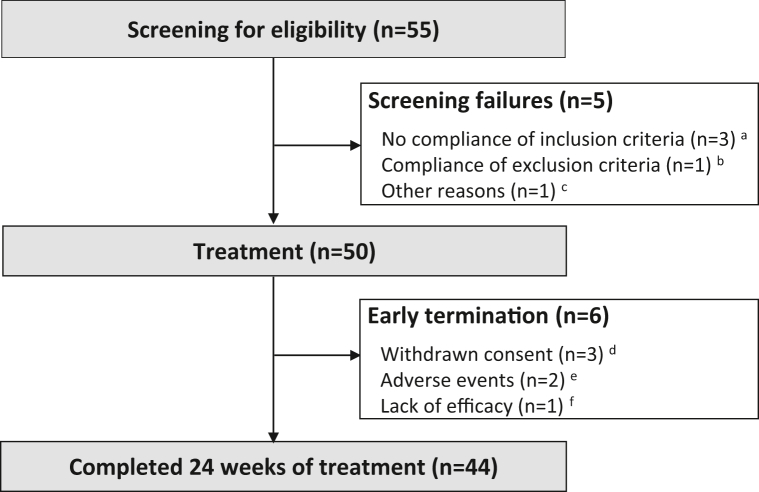
Patient disposition flowchart. A total of 55 patients were screened. **A,** Three patients did not meet the inclusion criteria: 2 patients had a palmoplantar pustulosis area and severity index (PPPASI) <12 at baseline and another patient had a serum creatinine ≥1.5 mg/dL. **B,** One patient met an exclusion criterion (severe hepatic dysfunction). **C,** One patient withdrew consent. Six patients terminated the study early: (**D**) Three patients have withdrawn the consent, one on visit 2, one on visit 3, and one on visit 4. **E,** Two patients discontinued the study because of the occurrence of treatment-emergent adverse events: 1 patient due to tinea pedis and worsening of palmoplantar pustulosis (PPP), the other one due to suspected drug reaction to guselkumab manifesting as nausea, vomiting, arthralgia, and injection-site reaction. **F,** One patient discontinued because of lack of efficacy.

In the scope of the GAP study, a total of 50 patients received guselkumab treatment at 8 German dermatological centers between September 2019 and July 2021 (Fig 1). Baseline characteristics of patients are summarized in Table I. Six patients terminated the study early (Fig 1).

**Table I:** Baseline characteristics of patients participating in the study

Characteristic	FAS, *n* = 50
Age in years, mean (SD)	56.9 (11.6)
Females, *n* (%)	41 (82.0%)
Body mass index, mean (SD)	28.2 (6.0)
Caucasians, *n* (%)	49 (98.0%)
Age at initial diagnosis of PPP in years, mean (SD)	46.1 (12.2)
Disease duration of PPP in years, mean (SD)	10.9 (11.7)
Concomitant psoriasis vulgaris, *n* (%)	29 (58.0%)
Concomitant psoriatic arthritis, *n* (%)	10 (20.0%)
Smoking status:	
Current smokers, *n* (%)	29 (58.0%)
Ex-smokers, *n* (%)	18 (36.0%)
Non-smokers, *n* (%)	2 (4.0%)
N.A. , *n* (%)	1 (2.0%)
PPPASI, mean (SD)	21.9 (7.8)
PASI, mean (SD), *n* = 29	2.0 (2.2)
DLQI, mean (SD)	14.2 (7.3)
Pustule count, mean (SD)	46.1 (47.7)
NRS pain, mean (SD)	6.2 (3.0)
NRS pruritus, mean (SD)	6.2 (2.9)
Patients with prior phototherapy∗	16 (32.0%)
UVA therapy, *n* (%)	2 (5.1%)
UVB therapy, *n* (%)	4 (10.3%)
PUVA therapy, *n* (%)	14 (35.9%)
Patients with prior systemic therapy§^,^†	27 (54.0%)
Methotrexate, *n* (%)	11 (28.2%)
Retinoids, *n* (%)	12 (30.8%)
Fumaric acid, *n* (%)	7 (17.9%)
Apremilast, *n* (%)	5 (12.8%)
Cyclosporine, *n* (%)	5 (12.8%)
Other‡, *n* (%)	11 (22.0%)

*DLQI*, Dermatology life quality index; *FAS*, full analysis set; *NRS*, numeric rating scale; *PPPASI*, palmoplantar pustulosis area and severity index; *PPSI*, palmoplantar pustulosis severity index; *PUVA*, psoralen plus ultraviolet A; *UVA*, ultraviolet A; *UVB*, ultraviolet B.

∗ Twelve patients had 1, 4 patients had 2 different prior phototherapies.

† Fourteen patients had 1, the other patients had 2 or more different prior systemic therapies.

‡ Other: Prednisolone: *n* = 1 (2.6%), Adalimumab: *n* = 2 (5.1%), Alefacept: *n* = 1 (2.6%), Infliximab: *n* = 1 (2.6%), Ixekizumab: *n* = 3 (7.7%), Onercept: *n* = 1 (2.6%), and Secukinumab: *n* = 2 (5.1%).

§ Information on previous therapies was available for 39 patients (78.0%).

**Fig 2:**
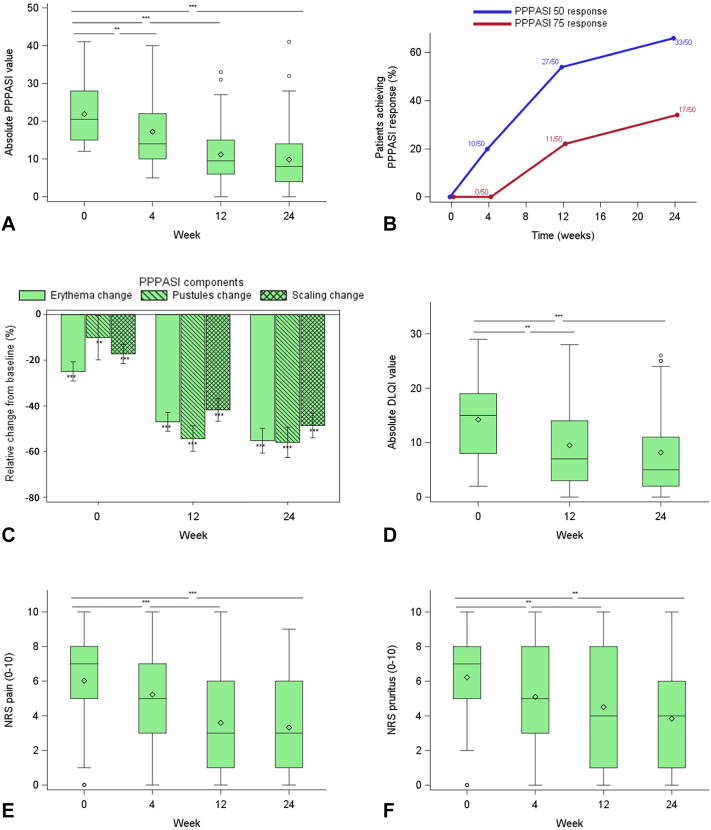
Guselkumab improves clinical scores and patient-reported outcomes in palmoplantar pustulosis (PPP) patients. **A,** Box-whisker plot of palmoplantar pustulosis area and severity index (PPPASI) values of guselkumab-treated patients (full-analysis-set population) over the study period (baseline [wk0], wk4, wk12, and wk24 [end of study]). Missing data were handled using baseline-observation-carried-forward (BOCF) method. The line in the *middle* of the box represents the median (second quartile, Q2), the *lower* and *upper* margins of the box represent the first (Q1) and third (Q3) quartile, respectively, and the ends of the whiskers represent the most extreme lower and upper values within Q3+1.5∗(Q3-Q1) and Q1-1.5∗(Q3-Q1), respectively). The *diamond* denotes the mean, *circles* denote outliers and *asterisks* denote a significant difference (∗∗∗: *P* < .001; ∗∗: *P* < .01) of PPPASI change from baseline. **B,** Proportions and absolute numbers of guselkumab-treated patients over the study period who achieved at least a 50% and a 75% improvement of the PPPASI. The BOCF method was used. **C,** Percentage changes (±SD) in individual skin alterations accounting for the PPPASI (degree of pustules, erythema, scaling) in guselkumab-treated patients at wk4, wk12, and wk24 relative to baseline. *Stars* indicate the significance of changes from baseline (∗∗∗: *P* < .001; ∗∗: *P* < .01). **D,** Box-whisker plot of absolute dermatology life quality index (DLQI) values of guselkumab-treated patients over the study period. The BOCF method was used. For description of box-whisper plots see (A). *Asterisks* denote a significant difference (∗∗∗: *P* < .001; ∗∗: *P* < .01) of the percent change from baseline. **E,** Box-whisker plot of pain values (determined by a numeric rating scale (NRS)] of guselkumab-treated patients over the study period. The BOCF method was used for description of box-whisper plots see (A). *Asterisks* denote a significant difference (∗∗∗: *P* < .001). **F,** Box-whisker plot of NRS pruritus values of guselkumab-treated patients over the study period. The BOCF method was used. For description of box-whisper plots see (A). *Asterisks* denote a significant difference (∗∗: *P* < .01).

The primary study endpoint was met with a median PPPASI reduction by 59.6% (first and third quartile [Q1/Q3]: 35.3%/85.7%) at wk 24 compared to baseline (*P* < .001). Significant PPPASI reduction was visible from wk 4 (Fig 2, *A*). The proportions of patients achieving PPPASI-50 and PPPASI-75 at wk 24 were 66.0% and 34.0%, respectively (Fig 2, *B*). Regarding the pustule count, there was a median reduction of 76.9% (Q1/Q3: 38.9%/100.0%; *P* < .001) at wk 24 compared to baseline. Clinical response did not show significant association with patient characteristics including smoking habit (wk 24 PPPASI change in current smokers versus nonsmokers: 61.5% [Q1/Q3: 27.3%/85.7%] versus 56.5% [Q1/Q3: 38.1%/69.2%]; *P* = .53; PPPASI-50 in current smokers versus nonsmokers: 69% versus 62%, *P* = .60) or concomitant PV (wk 24 PPPASI change in patients with versus without PV: 58.3% [Q1/Q3: 33.3%/86.7%] versus 61.5% [Q1/Q3: 48.3%/81.3%]; *P* = .71; PPPASI-50 in patients with versus without PV: 62% versus 71%, *P* = .49). Moreover, no impact of disease duration was observed (data not shown). It should be noted that the trial was not designed to evaluate the differences in response to guselkumab between smokers and nonsmokers or patients with and without concomitant PV. Over the 24 weeks, each of the 3 PPPASI components (erythema, pustules, and scaling) improved significantly (Fig 2, *C*).

**Fig 3:**
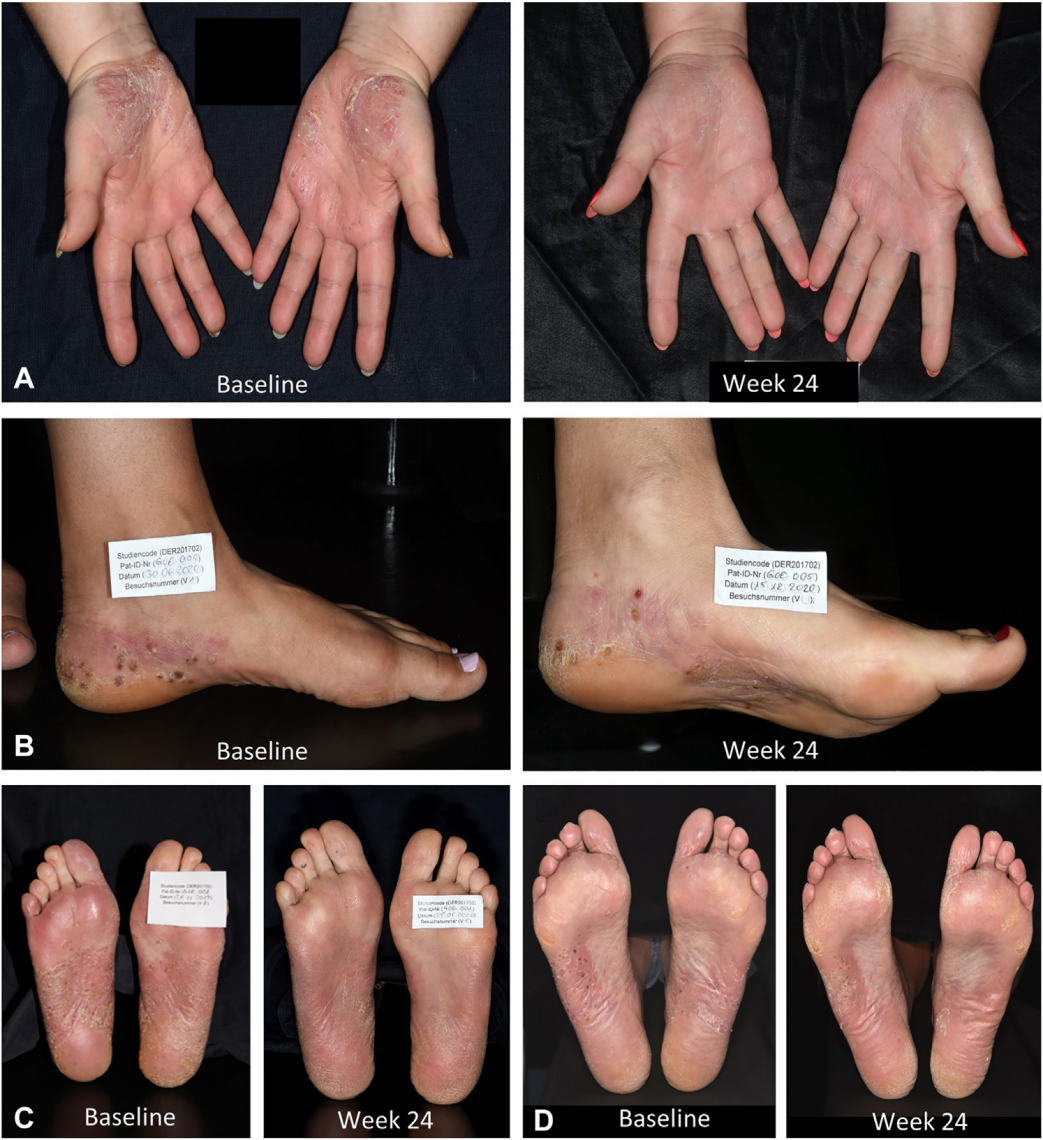
Clinical images and palmoplantar pustulosis area and severity index (PPPASI) of 4 representative patients at baseline and wk24. **A,** Palms of a 45-year-old female patient at baseline (PPPASI 22) and at wk24 (PPPASI 3). **B,** Plantae of a 52-year-old female patient at baseline (PPPASI 26) and at wk24 (PPPASI 11). **C,** Plantae of a 58-year-old female patient at baseline (PPPASI 35) and at wk24 (PPPASI 16). **D,** Plantae of a 59-year-old male patient at baseline (PPPASI 17) and at wk24 (PPPASI 2).

The dermatology life quality index decreased from a median of 15.0 (Q1/Q3: 8.0/19.0) at baseline to 5.0 (Q1/Q3: 2.0/11.0) at wk 24 (Fig 2, *D*). Numeric rating scale pain declined from median 7.0 (Q1/Q3: 5.0/8.0) at baseline to 4.0 (Q1/Q3: 1.0/6.0) at wk 24 (Fig 2, *E*). Numeric rating scale pruritus dropped from a baseline median of 7.0 (Q1/Q3: 5.0/8.0) to 4.0 (Q1/Q3: 2.0/7.0) at wk 24 (Fig 2, *F*). Clinical pictures of 4 representative treatment courses are shown in Fig 3, *A*-*D*.

**Fig 4:**
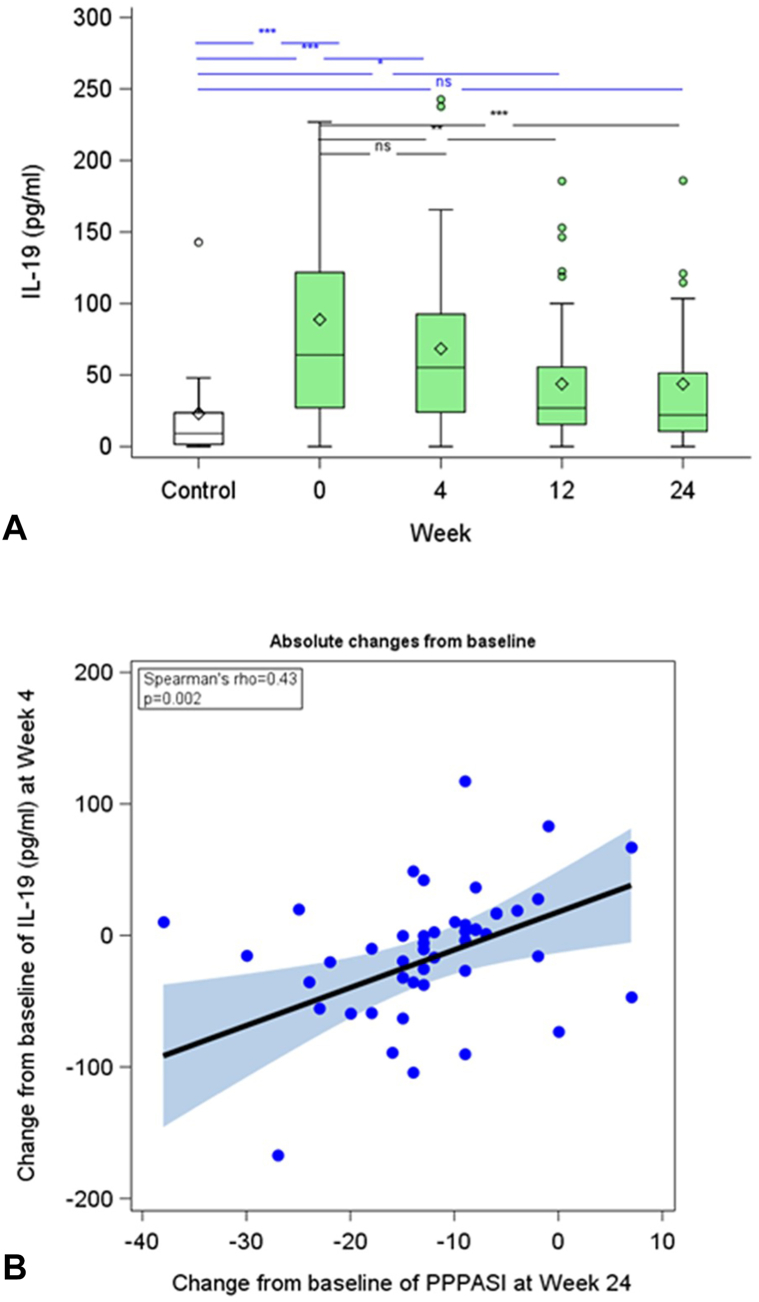
The interleukin (IL)-19 serum level may be a predictive biomarker for the treatment response of PPP patients to guselkumab. **A,** Box-whisker plot of absolute IL-19 serum levels of guselkumab-treated PPP patients (full-analysis-set population; missing values have not been imputed; wk0 (baseline): *n* = 50, wk4: *n* = 48, wk12: *n* = 47, and wk24: *n* = 44) and 14 healthy control participants. The line in the *middle* of the box represents the median (second quartile, Q2), the *lower* and *upper* margins of the box represent the first (Q1) and third (Q3) quartile, respectively, and the ends of the whiskers represent the most extreme lower and upper values within Q3+1.5∗(Q3-Q1) and Q1-1.5∗(Q3-Q1), respectively). The *diamond* denotes the mean and *circles* denote outliers. *Asterisks* denote a significant difference compared to heathy control participants (*blue*) or study patients at wk0 (*black*) (∗∗∗: *P* < .001, ∗∗: *P* < .01, ∗: *P* < .05; ns: not significant). **B,** Correlation between the absolute changes in IL-19 serum level between wk4 and baseline versus the absolute changes in PPPASI between wk24 and baseline in guselkumab-treated patients (missing values have not been imputed, *n* = 43). Spearman correlation coefficient and *P* value are shown.

Serum levels of IL-19 in PPP patients were found to be significantly higher at baseline when compared to levels in healthy control participants (mean ± standard deviation: 88.8 ± 92.1 pg/ml vs 23.0 ± 37.2 pg/ml, *P* < .001) (Fig 4, *A*). Baseline IL-19 levels in PPP patients showed a positive correlation with PPPASI (r_s_ = 0.55, *P* < .001) and were independent of patients’ age, gender, body mass index, and disease duration (data not shown).

Treatment of PPP patients with guselkumab resulted in a reduction in IL-19 levels compared to baseline, reaching significance at wk 12 and wk 24 (Fig 4, *A*). Moreover, the reduction in IL-19 serum levels at wk 4 correlated with the reduction in PPPASI at wk 24 (r_s_ = 0.41, *P* < .01) (Fig 4, *B*) and also with the reduction in pustule count at wk 24 (r_s_ = 0.39, *P* < .05; data not shown).

### Safety assessments

A total of 102 treatment-emerged adverse events (TEAEs) occurred in 37 patients and were mainly mild to moderate in severity. Seventeen TEAEs (16.7%) were assessed by the investigators to be related to guselkumab treatment. Most frequent TEAEs were nasopharyngitis (in 5 patients), and arthralgia (in 4 patients). Four serious adverse events (ruptured ectopic pregnancy, chronic cholecystitis, cholecystectomy, and peritonitis) were reported in 2 of the 50 patients and were assessed to be unrelated to guselkumab treatment by the investigators. Two TEAEs led to discontinuation of treatment in 2 patients. No deaths were reported during the study.

## Discussion

This phase-II clinical trial met its primary endpoint with a significant improvement of the PPPASI at wk 24. Guselkumab treatment was also effective in reducing the investigated patient-reported outcome measures. With 98% of the participants being Caucasian, this is the first clinical trial showing therapeutic effect of guselkumab in this population. The parameters that may be compared between our study and the studies conducted in Japan^27^, ^28^, ^29^ indicate similar efficacy of guselkumab in European and Japanese patients, exceeding that of placebo (PPPASI-50/-75: guselkumab/Caucasian: 66.0%/34.0%, guselkumab/Japanese: 57.4%/20.4%, placebo/Japanese: 34.0%/3.8%).^27^ In our study, safety findings were comparable to guselkumab studies in PV,^30^ with no new safety concerns.

In contrast to studies analyzing the efficacy of methotrexate, cyclosporine A, and acitretin, some studies investigating the efficacy of other biologics in patients with PPP involved larger numbers of patients, adequate treatment duration, and outcomes also used in our study (PPPASI-50 and/or PPPASI-75), and may therefore allow a rough comparison with our study results.^24^^,^^26^ Of all biologics studied so far, guselkumab appears to have the highest efficacy. For example, the IL-17A inhibitor secukinumab appears to be less effective in Caucasian patients with PPP. In the phase-3b study conducted in Europe, only 27% of PPP patients treated with 300 mg secukinumab reached PPPASI-75 at wk 16 (the primary endpoint of the study not met).^24^ This suggests IL-23-dependent, and, in addition to IL-17A-dependent, also IL-17A-independent mechanisms in PPP pathogenesis. The observed efficacy of guselkumab was clearly higher than that achieved in the randomized placebo-controlled trial with ustekinumab (PPPASI-50 at wk 16: 10%),^23^ an inhibitor of both IL-23 and IL-12, suggesting a protective effect of the IL-12-dependent pathway in PPP. Furthermore, spesolimab, an anti-IL-36 receptor antibody approved for the treatment of generalized pustular psoriasis, was also less effective in patients with PPP (PPPASI-50 at wk 16: 32% of spesolimab-treated versus 23.8% of placebo-treated patients; primary study endpoint not met).^26^

The results of our study therefore indicate that IL-23 is a key mediator of PPP. Most likely, IL-23 does not directly induce local infiltration of neutrophilic granulocytes into the skin or alter growth and differentiation of keratinocytes, that is, alterations that become clinically visible as pustules and scaly thickening, respectively. Instead, several cytokines produced by IL-23-stimulated cells or even more downstream mediators may mediate these alterations.^31^ One of the mediators downstream of IL-23 that has been proposed to be causally involved in the persistence of pustules in PPP, is IL-19.^14^ IL-19 is known to be produced by keratinocytes activated with T-cell cytokines such as IL-4, IL-17A, and IL-22.^32^, ^33^, ^34^ Furthermore, there is a positive feedback loop in PPP, with IL-19 also being secreted by neutrophils and supporting keratinocytes to increase their production of the neutrophil-attracting chemokine CXCL6.^14^ Interestingly, a T17-to-T2 cell transition with increase in IL-23 receptor expression on these cells has been suggested for the T cells in PPP lesions.^17^ Interferon-gamma (IFN-γ), a key cytokine of the IL-12 pathway, may have a negative impact on these cells.^35^^,^^36^ Of note, in addition to the induction of IL-19 and other soluble mediators, IL-22 and IL-4 directly alter the differentiation of keratinocytes and may therefore be responsible for the thickening and scaling of PPP lesions.^8^^,^^31^ Interestingly, increased IL-19 serum levels and the predictive value of early changes in IL-19 serum levels for the response to guselkumab treatment observed in our current study imply that this mediator is a potential biomarker in PPP.

Limitations of our study design include the absence of a placebo group and the short observation time of 24 weeks. It also should be noted that comparing the efficacy of different medical drugs in PPP has limitations, especially as head-to-head studies are lacking and as existing studies frequently involved small numbers of patients and different efficacy parameters.

In conclusions, the results of this study indicate that guselkumab represents an effective therapeutic option with a favorable safety profile for PPP patients also within the Caucasian population. Our study further suggests the suitability of IL-19 serum levels as an early predictor of the therapeutic response in PPP.

## Conflicts of interest

Dr Wilsmann-Theis has been an advisor, speaker, or investigator for Abbvie, Almirall, Amgen, Biogen, Boehringer Ingelheim, Bristol Myers Squibb, Celgene, GlaxoSmithKline, Hexal, Incyte, Janssen-Cilag, Leo Pharma, Eli Lilly, Medac, Merck Sharp & Dohme Corp., Novartis, Pfizer, and UCB Pharma. Author Patt has been investigator for and/ or received grants from AbbVie, AnaptysBio, Boehringer Ingelheim, Bristol-Myers Squibb, Celgene, Galderma, Incyte, Janssen, LEO Pharma, Novartis Pharma, OM Pharma, Pfizer, Regeneron, and UCB Pharma. Dr Pinter has served as an advisor and/or paid speaker for and/or participated in clinical trials sponsored by: AbbVie, Almirall-Hermal, Amgen, Biogen Idec, Biontec, BMS, Boehringer-Ingelheim, Celgene, Celltrion, GSK, Eli-Lilly, Eva Pharma, Galderma, Hexal, Incyte, Janssen-Cilag, Klinge Pharma LEO-Pharma, MC2, Medac, Merck Serono, Mitsubishi, Moonlake, MSD, Novartis, Pascoe, Pfizer, Tigercat Pharma, Regeneron, Roche, Sandoz Biopharmaceuticals, Sanofi-Genzyme, Schering-Plough, UCB Pharma, and Zuellig Pharma. Dr Gerdes has been an advisor and/or received speakers' honoraria and/or received grants and/or participated in clinical trials of the following companies: AbbVie, Acylering, Affibody AB, Akari Therapeutics Plc, Almirall-Hermal, Amgen, Anaptys Bio, Argenx BV, AstraZeneca AB, Bioskin, Bristol-Myers Squibb, Boehringer-Ingelheim, Celgene, Dermira, Eli Lilly, Galderma, Hexal AG, Incyte Inc., Janssen-Cilag, Johnson & Johnson, Klinge Pharma, Kymab, Leo Pharma, Medac, Neubourg Skin Care GmbH, Novartis, Pfizer, Principia Biopharma, Regeneron Pharmaceutical, Sandoz Biopharmaceuticals, Sanofi-Aventis, and UCB Pharma. N Magnolo has received honoraria as an advisor, speaker, and/or consultant AbbVie, Almirall, Amgen, Boehringer Ingelheim, Bristol Myers Squibb, Celgene, Janssen-Cilag, La Roche-Posay, LEO Pharma, Lilly, Novartis, Pfizer, Dr Wolff, and UCB Pharma. Drs Németh, Paul, Hüffmeier has no conflict of interest to declare. Author Schmitz has no conflict of interest to declare. Dr Paul has no conflict of interest to declare. Dr Augustin has served as a consultant, lecturer or researcher, and/or has received research grants from companies manufacturing drugs for psoriasis, including AbbVie, Almirall, Amgen, Biogen, Boehringer Ingelheim, BMS, Celgene, Centocor, Eli Lilly, Galderma, Hexal, Janssen, Klinge, LEO, Medac, MSD, Mylan B.V., Novartis, Pfizer, Sandoz, Takeda, UCB, and Viatris. Dr Staubach has received research grants, travel grants, consulting or lecturer's honoraria from Abbvie, Allergika, Almirall-Hermal, Amgen, Avene, Unna Akademie, Biocryst, BMS, Boehringer-Ingelheim, Celgene, CSL-Behring, Eli-Lilly, Galderma, GSK, Janssen, Klinge, LEO-Pharma, L'Oreal, Novartis, Octapharma, Pfizer, Pharming, Regeneron, Shire, Takeda, Sanofi-Genzyme, and UCB Pharma. Dr Weyergraf has served as a speaker, advisor and/or researcher for AbbVie, Almirall, Amgen, Arctic Bioscience, Biogen, Bristol-Myers-Squibb, Celgene, Hermal, Janssen, LEO, Lilly, Novartis, Pfizer, Sanofi, and UCB. Dr Wolk has received research grants or contracts for clinical trials (payment to her institution), support for attending congresses, scientific awards, consulting fees or honoraria for participation in advisory boards, or honoraria for lectures for one or more of the following: Celgene/Amgen, Celgene/Bristol Myers Squibb, Charité Research Organization, Flexopharm, Janssen-Cilag, Novartis Pharma, Sanofi–Aventis, TFS Trial Form Support, University hospital Magdeburg, European HS foundation (EHSF), and the Symposium on Hidradenitis Suppurativa Advances (SHSA); she also has an non-financial relationship to the HS task force of the German Consortium for Dermatological Research (ADF). Dr Sabat has received research grants, clinical trial contracts, scientific awards, or honoraria for consulting, participation in advisory boards, or for lectures for one or more of the following: AbbVie, Almirall Hermal, Amgen, Bayer Schering Pharma, Boehringer Ingelheim Pharma, Bruno Bloch Stiftung, Celgene/Amgen, Celgene/Bristol Myers Squibb, Charité Research Organisation, CSL Behring, ICON, IQVIA RDS, Incyte, Janssen-Cilag/Janssen Research & Development, MoonLake Immunotherapeutics, Novartis Pharma, Parexel, Rheinischen Friedrich-Wilhelms-Universität Bonn, Sanofi–Aventis, TFS, UCB Biopharma, Universitätsmedizin Greifswald, and Wundnetz Berlin-Brandenburg e. V. Dr Mӧßner has been an advisor and/or received speakers’ honoraria and/or received grants and/or participated in clinical trials of the following companies: Abbvie, Allmirall, Biogen IDEC GmbH, Böhringer-Ingelheim, Celgene, Janssen-Cilag GmbH, Leo Pharma GmbH, Eli Lilly and Company, Merck Serono GmbH, MSD SHARP & DOHME GmbH, Novartis Pharma GmbH, Pfizer GmbH, and UCB.
